# Proposing a machine-learning based method to predict stillbirth before and during delivery and ranking the features: nationwide retrospective cross-sectional study

**DOI:** 10.1186/s12884-021-03658-z

**Published:** 2021-03-12

**Authors:** Toktam Khatibi, Elham Hanifi, Mohammad Mehdi Sepehri, Leila Allahqoli

**Affiliations:** 1 School of Industrial and Systems Engineering, Tarbiat Modares University (TMU), Tehran, 14117-13114 Iran; 2 Endometriosis Research Center, Iran University of Medical Sciences (IUMS), Tehran, Iran

**Keywords:** Stillbirth prediction, Classification, Feature selection, Ensemble learning, IMAN registry

## Abstract

**Background:**

Stillbirth is defined as fetal loss in pregnancy beyond 28 weeks by WHO. In this study, a machine-learning based method is proposed to predict stillbirth from livebirth and discriminate stillbirth before and during delivery and rank the features.

**Method:**

A two-step stack ensemble classifier is proposed for classifying the instances into stillbirth and livebirth at the first step and then, classifying stillbirth before delivery from stillbirth during the labor at the second step. The proposed SE has two consecutive layers including the same classifiers. The base classifiers in each layer are decision tree, Gradient boosting classifier, logistics regression, random forest and support vector machines which are trained independently and aggregated based on Vote boosting method. Moreover, a new feature ranking method is proposed in this study based on mean decrease accuracy, Gini Index and model coefficients to find high-ranked features.

**Results:**

IMAN registry dataset is used in this study considering all births at or beyond 28th gestational week from 2016/04/01 to 2017/01/01 including 1,415,623 live birth and 5502 stillbirth cases. A combination of maternal demographic features, clinical history, fetal properties, delivery descriptors, environmental features, healthcare service provider descriptors and socio-demographic features are considered. The experimental results show that our proposed SE outperforms the compared classifiers with the average accuracy of 90%, sensitivity of 91%, specificity of 88%. The discrimination of the proposed SE is assessed and the average AUC of ±95%, CI of 90.51% ±1.08 and 90% ±1.12 is obtained on training dataset for model development and test dataset for external validation, respectively. The proposed SE is calibrated using isotopic nonparametric calibration method with the score of 0.07. The process is repeated 10,000 times and AUC of SE classifiers using random different training datasets as null distribution. The obtained *p*-value to assess the specificity of the proposed SE is 0.0126 which shows the significance of the proposed SE.

**Conclusions:**

Gestational age and fetal height are two most important features for discriminating livebirth from stillbirth. Moreover, hospital, province, delivery main cause, perinatal abnormality, miscarriage number and maternal age are the most important features for classifying stillbirth before and during delivery.

**Supplementary Information:**

The online version contains supplementary material available at 10.1186/s12884-021-03658-z.

## Background

Stillbirth is defined as fetal loss in pregnancy beyond 28 weeks by World Health Organization. It is a major public health problem in all countries [1] specially in low resource regions [2]. According to WHO report, 2.6 million stillbirths have been occurred globally in 2015 which 98% has been reported in low- and middle-income countries [3].

Importance and necessity of stillbirth prediction and identifying its main risk factors can be helpful to prevent from stillbirth occurrence or reduce its probability in many high risk cases. It could reduce the burden of stillbirth. Therefore, predicting stillbirth from different maternal and prenatal features has been an attractive and important research topic in the previous studies [4–7].

Some previous studies have focused on early prediction of stillbirth in the first trimester of pregnancy or before 14th to 25th gestational week [1, 4, 8]. But, a half of stillbirth cases have been occurred during the labor based on WHO report [3]. Therefore, the main aim of this study is not early prediction of stillbirth. But instead, the research problem in this study is to predict stillbirth before and during delivery with considering the maternal demographic characteristics, clinical features, fetal properties, labor descriptors, environmental features, healthcare service provider descriptors and socio-demographic features as the input variables. Moreover, the input features are ranked and scored based on their predictive power for determining different types of stillbirth cases. For this purpose, in this study, a big dataset including about 1,431,597 birth cases which is registered in Iranian Maternal and neonatal (IMAN) registry from 2016/04/01 to 2017/01/01 is analyzed using several machine learning methods.

The rest of the paper is organized as follows: Section 2 states related works for stillbirth prediction. In Section 3, the methodology of this research is described and then the results and findings are reported in Sect. 4. Section 5 discusses about the main findings of this study. Concluding remarks are presented in Sect. 6.

## Related works

In this section, we will review the previously related studies for stillbirth prediction. For this purpose, the related works are considered from several aspects including the considered features, the analytical methods for stillbirth prediction and the analyzed dataset characteristics. More details of related works are described in Additional file 1.

Different characteristics and features have been analyzed in the previous studies for stillbirth prediction such as maternal age [9], obesity [9], birth weight [7, 10] and fetal growth restrictions [2, 7, 11], having prior stillbirth [7], race [7], maternal comorbidities [2, 11, 12], maternal occupation [11], parity [11], bleeding in pregnancy [11], fetal anatomical properties [4], maternal life style descriptors [12] and socio-demographic features [6].

The previous studies have used univariate [5] and/or multivariate statistical analysis [2, 13] and machine learning classifiers such as logistics regression (LR) [4, 6, 11, 13, 14], decision trees (DT), random forest, extreme gradient boosting (XGBoost), and a multilayer perceptron neural network (MLP) [6] for stillbirth prediction.

A main advantage of our study compared to the previous studies is analyzing the large population (all stillbirth and living birth cases registered in Iranian Maternal and neonatal records (IMAN registry) from 2016/04/01 to 2017/01/01. In this study, all births at or beyond 28th gestational week are considered including 1,415,623 live birth and 5502 stillbirth cases which 4557 and 144 cases are occurred before delivery and during it, respectively. Time of stillbirth for 801 cases is unknown, therefore, they are excluded from the second step of the study which classifies stillbirth cases into before delivery and during it. To the best of our knowledge, our analyzed dataset is one of the biggest datasets considered in the studies for stillbirth prediction till now. Moreover, different types of stillbirth (before delivery and during the labor process) are classified, too.

Most of the previous studies have focused on stillbirth prediction during the first-trimester of the pregnancy. But, according to the report published by World Health Organization, about 50% of stillbirths have been occurred during the labor [3]. Therefore, the characteristics of the labor may be some risk factors leading to stillbirth for significant ratio of stillbirth cases. Thus, the aim of this study is not early prediction of stillbirth. But instead, we want to assess the effect of the labor properties as the late predictors of stillbirth to discriminate livebirth from stillbirth and classify stillbirth cased into before and during delivery classes.

In this study, several machine learning classifiers are trained and used for stillbirth prediction to reach the best accuracy. Each classifier used in this study has different capabilities. To the best of our knowledge, our used machine learning classifiers are many more than the previously used classifiers for stillbirth prediction.

Moreover, different and more features are considering in this study for stillbirth prediction. On the other hand, the significance and importance of each feature is measured using several machine learning methods and finally, features are ranked based on their discriminative power to predict stillbirth and living birth cases.

The main novelties of this study lies in several folds including:

- To the best of our knowledge, our dataset is one of the biggest datasets considered yet for stillbirth prediction. The dataset is collected from all regions of Iran country from 2016/04/01 to 2017/01/01.
- Classifying data into livebirth, stillbirth before delivery and stillbirth during delivery.
- A new combination of the features is considered in this study for stillbirth prediction.
- A novel feature ranking method combining different feature importance measures is proposed and used in this study for ranking the predictive features for stillbirth prediction.
- A novel stack ensemble classifier is designed and proposed in this study for stillbirth prediction.
- The proposed novel stack ensemble outperforms the compared classifiers for predicting stillbirth cases in our dataset.

## Methods

**Fig. 1:**
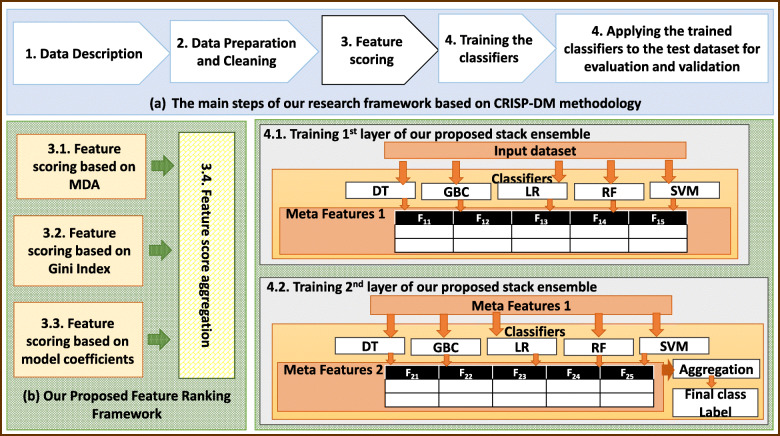
The main steps of our proposed research method for stillbirth prediction and feature ranking (MDA: Mean Decrease of Accuracy, DT: Decision Tree, GBC: Gradient Boosting Classifier, LR: Logistics Regression, RF: Random Forest, SVM: Support Vector Machines, A.R.: Aggregation Rule)

The main steps of our proposed research method for stillbirth prediction and feature ranking is shown in Fig. 1:

As illustrated by Fig. 1(a) more details of the steps of our proposed method which is based on Cross-industry standard process for data mining (CRISP-DM) methodology as the popular standard framework for data analytics application will be described in the following subsections. Figure 1(b) indicates the architecture of our proposed feature ranking framework and Fig. 1 (c) shows the architecture of our proposed stack ensemble method for classifying the instances.

### Data description

In this study, a big dataset including about 1,431,597 birth cases which is registered in Iranian Maternal and neonatal (IMAN) registry from 2016/04/01 to 2017/01/01 is analyze. In this study, all births at or beyond 28th gestational week are considered from which 5602 stillbirth and 1,415,623 live birth cases are occurred, respectively. From stillbirth cases, 4557 and 144 cases are occurred before and during delivery, respectively. The exact time of stillbirth for 901 cases is not known and they will be excluded from the second step classification in which stillbirth before delivery is discriminated from stillbirth during delivery. Therefore, in our dataset, 0.396% of birth cases are stillbirth. Table 1 shows the features in our dataset for stillbirth prediction.

**Table 1:** list of the features considered in our dataset for stillbirth prediction (F.Code: Feature Code, M: Maternal, P: Perinatal, E: Enviromental, H: Health)

F.Code	M/P/E/H	Feature	Feature Type
f1	M	any pregnancy risk factor	Binary
f2	M	gestational diabetes	Binary
f3	M	cardiovascular diseases	Binary
f4	M	other maternal underlying diseases	Binary
f5	M	chronic hypertension	Binary
f6	P	fetal abnormalities	Binary
f7	M	Human immunodeficiency virus (HIV+)	Binary
f8	M	Venereal Disease Research Laboratory test (VDRL+)	Binary
f9	M	preeclampsia or eclampsia risk factors	Binary
f10	P	Intrauterine growth restriction (IUGR)	Binary
f11	M	infant mortality after previous deliveries	Binary
f12	M	occurring stillbirth in the previous pregnancies	Binary
f13	M	Type1 or Type 2 diabetes	Binary
f14	M	hepatitis B	Binary
f15	M	Chorioamnionitis	Binary
f16	M	maternal drug or alcohol addiction	Binary
f17	M	smoking	Binary
f18	P	placental abruption	Binary
f19	P	Meconium-stained amniotic fluid	Binary
f20	P	Irregular fetal heartbeat	Binary
f21	P	Early rupture of the amniotic sac	Binary
f22	M	risks or dangerous causes of delivery	Nominal
f24	M	maternal autoimmune disease	Binary
f25	M	epidural anesthesia	Binary
f26	P	Placenta accreta	Binary
f27	M	IVF in the current pregnancy	Binary
f28	M	number of the pregnancies	Numeric
f29	M	number of the previous deliveries	Numeric
f30	M	miscarriage number	Numeric
f31	P	gestational age	Numeric
f32	P	fetal weight (grams)	Numeric
f33	M	birth number	Numeric
f34	M	number of live children from the previous pregnancies	Numeric
f35	M	type of delivery (natural delivery or cesarean)	Binary
f36	M	cesarean main cause	Nominal
f37	H	medical science university operator the hospital	Nominal
f38	E	province	Nominal
f39	H	hospital	Nominal
f40	H	hospital type	Nominal
f41	P	fetal gender	Nominal
f42	M	delivery main cause	Nominal
f43	H	delivery place	Nominal
f44	M	maternal nationality	Nominal
f45	M	maternal education	Ordinal
f46	M	Consanguinity with spouse	Binary
f47	E	city	Nominal
f48	M	maternal insurance type	Nominal
f49	P	fetal height (cm.)	Numeric
f52	M	father nationality	Nominal
f53	M	maternal age	Numeric
Outcome1	–	outcome of the pregnancy: stillbirth or livebirth	Binary
Outcome2	–	stillbirth before delivery process, stillbirth during delivery process, unknown	Nominal

As shown in Table 1, M/P/E/H denotes the feature describes the maternal (M), perinatal (P), environment (E) and health system (H) related factors.

This study aims at a hierarchical classification process in which data instances are classified into stillbirth and livebirth classes based on outcome1 feature at first. Then, the instances assigned stillbirth class label are classified again into before delivery and during delivery classes based on outcome2 feature. Stillbirth cases having unknown occurrence time are excluded from the second step classification.

**Fig. 2:**
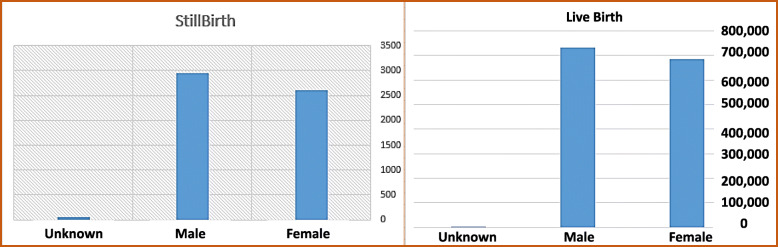
The frequency of stillbirth and livebirth based on fetal gender

Figure 2 denotes the frequency of stillbirth and livebirth based on fetal gender.

As shown by Fig. 2, the highest ratio of stillbirth to live birth occurs for fetal having unknown gender. Moreover, the ratio of stillbirth to livebirth for males is higher than for females.

**Fig. 3:**
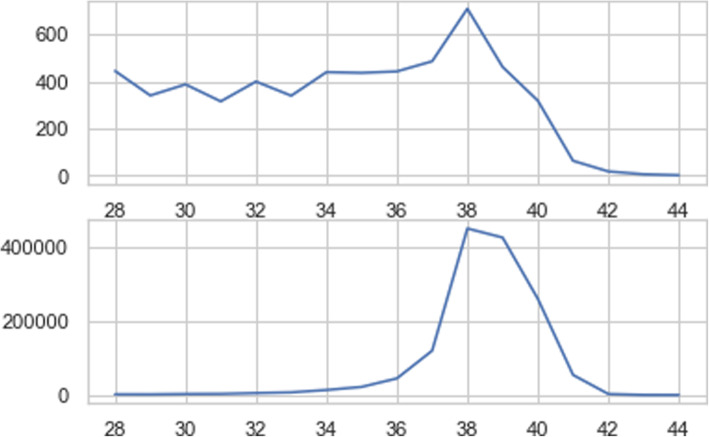
Stillbirth distribution based on gestational age

Figure 3 indicates stillbirth and live birth frequency in terms of gestational age.

As depicted in Fig. 3, stillbirth distribution differs significantly from livebirth in terms of gestational age. Therefore, gestational age can be a good predictor to discriminate stillbirth from livebirth. Moreover, the relationship between stillbirth frequency and gestational age is not a linear relationship and some fluctuations exist from 28th to 34th gestational age. The number of stillbirth cases is approximately similar for 34th, 35th and 36th gestational age but the number of livebirth cases increases with a dramatic slope from 34th to 38th gestational week. The peak of the livebirth frequency occurs in 38th gestational week and the number of livebirth cases before 34th gestational week is very low.

The frequency of stillbirth falls widely after 38th gestational week but its frequency in 39th gestational week is not very fewer than 28 to 33th gestational week. The number of stillbirth cases reduces significantly in 40th gestational week comparing to the previous weeks.

**Fig. 4:**
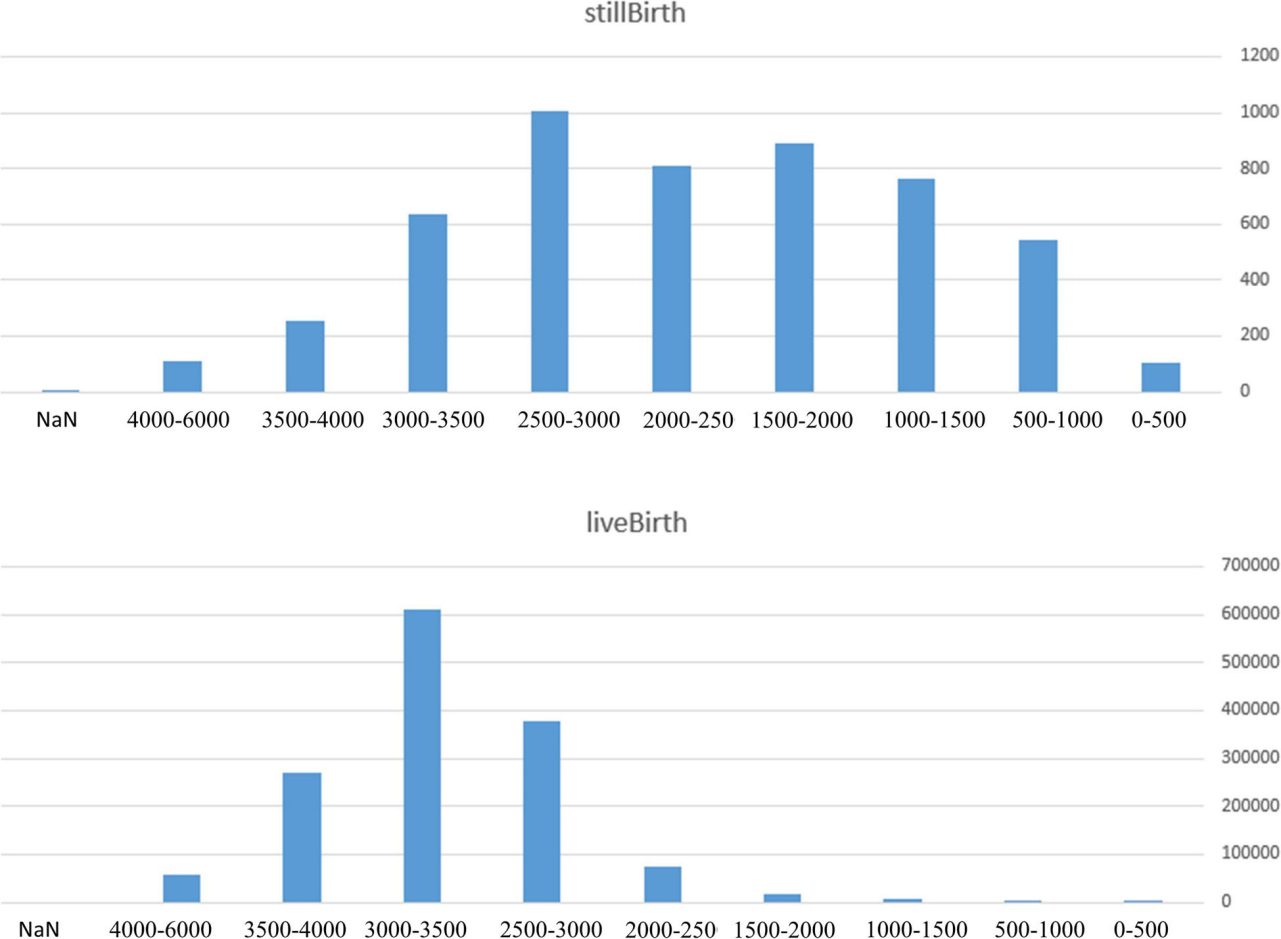
The number of stillbirth and livebirth based on fetal weight (gr)

Figure 4 depicts the number of stillbirth and livebirth in terms of fetal weight (grams).

As shown by Fig. 4, the distribution of stillbirth and livebirth based on fetal weight differs significantly. It may show that fetal weight is a good feature to discriminate stillbirth and livebirth cases.

The average ± standard deviation of the number of pregnancies for livebirth and stillbirth cases is 2.61 ± 1.67 and 3.05 ± 1.92, respectively. The average ± standard deviation of the gestational age for livebirth and stillbirth cases is 38.53 ± 1.57 and 34.95 ± 3.99, respectively.

The maternal age has the average ± standard deviation of 27.98 ± 6.27 and 29.73 ± 6.73 for livebirth and stillbirth cases, respectively. Birth weight for livebirth and stillbirth cases has the average ± standard deviation of 3122.90 ± 485.65 and 2198.246 ± 939.84, respectively. Missing value rate for the features considered in this study lies between 0 and 4.63%. Features having more than 5% missing values are removed from the study. The 19.74% of the population have missing values.

### Data preprocessing and cleaning

Since our dataset is a real dataset registered in IMAN registry, it should be preprocessed and cleansed to improve its quality for further analytical purposes. The main steps of data preprocessing and cleaning are data sampling, nominal variable conversion, missing value imputation and normalization which will be described in more details in Additional file 1.

### Feature ranking

A new feature ranking method is proposed in this study which is shown by Fig. 1 (b). Our proposed feature ranking method consists of four different methods for measuring the feature importance and a module for aggregating the feature scores to generate the final feature score.

Feature importance in our proposed method is measured based on mean decrease accuracy (MDA), Gini Index (GI) and model coefficients (MC) which their equations are shown in Additional file 2.

MC denotes the classifier coefficients if it is available. For example, SVM with linear kernels finds the coefficients of different input features of the hyperplane equation separating data instances of two classes.

Some previous studies have used SVM for feature scoring, too [15]. Feature scores can be determined by the absolute values of the feature coefficients of the hyperplane separating two different classes in SVM with linear kernel. SVM coefficients of the features show which features are more important and which ones are not important.Before aggregating feature scores generated with different measures and different classifiers, the feature scores are concatenated column-wise to produce meta feature table (MFT) as shown in Table 2.

**Table 2:** meta feature table (MFT) listing feature scores generated with different measures and classifiers (FS: Feature Score, MDA: Mean Decrease of Accuracy, IG: Gini-Index, MC: Model Coefficients)

Feature	FS (Classifier1,MDA)	FS (Classifier1,GI)	FS (Classifier1,MC)	…	FS (ClassifierS,MC)	…
F_1_	Score_1,1_	Score_1,2_	Score_1,3_	…	Score_1,q_	…
F_2_	Score_2,1_	Score_2,2_	Score_2,3_	…	Score_2,q_	…
…	…	…	…	…	…	…
F_p_	Score_p,1_	Score_p,2_	Score_p,3_	…	Score_p,q_	…
…	…	…	…	…	…	…

As listed in Table 2, F_p_ denotes the p^th^ input feature, FS (Classifier_s_, X) denotes the feature score for the features obtained by the s^th^ classifier based on X measure. X measure can be MDA, IG, MC and CN. Score_p,q_ denotes the score of the p^th^ feature obtained by the q^th^ feature scoring method.

As illustrated by Table 2, each column of MFT indicate the corresponding feature score generated with a feature importance measure and a classifier. Each row of MFT is corresponding to one input feature of our dataset for stillbirth prediction.

Every column of MFT is normalized with min-max normalization method to avoid dominating any column by another one because of having different range of values.

Rows of MFT are clustered using K-means as a popular, simple and fast clustering method. K-means partitions data instances into K non-overlapping convex-shaped clusters. Each data instance should be a member of exactly one cluster in K-means clustering method. K-means has some challenges including initializing the first-round cluster centroids, determining the number of clusters as a hyper-parameter of K-means and determining the appropriate distance function [16]. Several solutions have been proposed to overcome or address the mentioned challenges [16]. In this study, initialization of the cluster centroids is done by choosing some data instances are the initial centroids by random. Distance function used in this study for data clustering is the Euclidean distance function because of its appropriateness for MTF which all columns have numerical type.

But, for determining the best number of clusters (K), the clustering validity index named Silhouette is used. Silhouette is one of the clustering validity indices which is used for clustering interpretation and validation by measuring the quality of the clustering results. Silhouette is calculated based on intra cluster tightness and inter cluster separation.

Silhouette lies between − 1 and 1 and nearest values to 1 shows better clustering result.

**Fig. 5:**
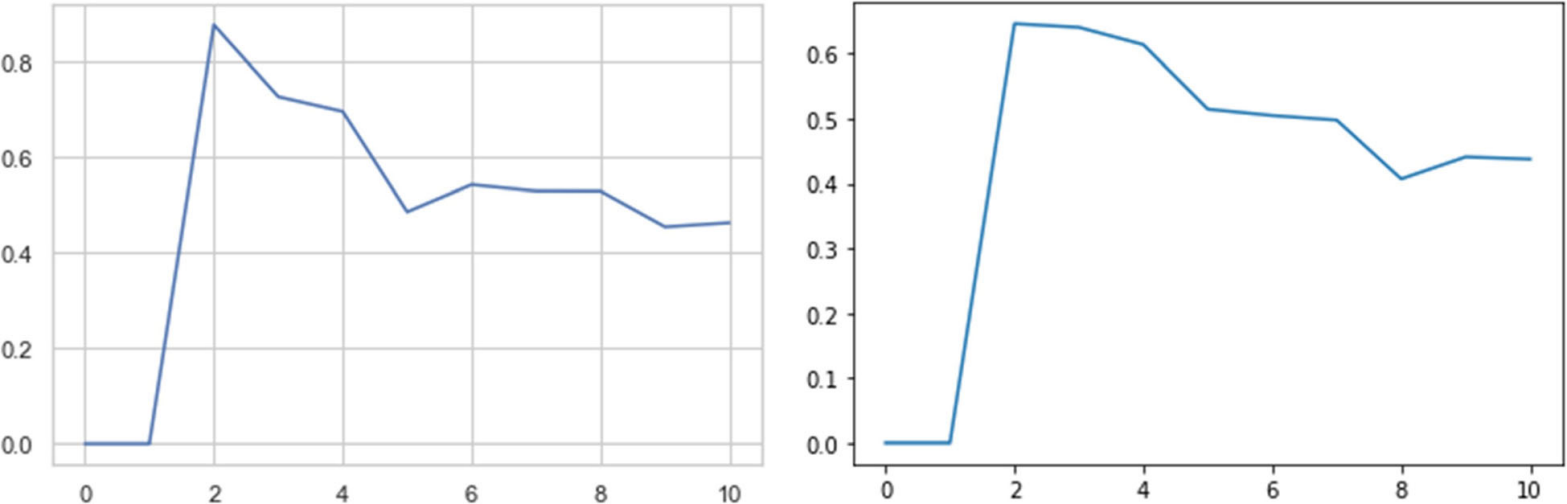
The average silhouette measure for clustering resulted by K-means for different number of clusters (left: first step classification task, right: second step classification task)

In this study, K is changed from 2 to 10 clusters and the average silhouette is calculated for each clustering resulted by K-means as shown in Fig. 5.

As shown by Fig. 5, better clustering results are obtained for 2, 4 and 6 clusters for the first-step classification and 2, 3 and 7 clusters for the second-step classification tasks. Therefore, we consider the results of K-means for K = 2, 4 and 6 for classifying the instances into livebirth and stillbirth classes and K = 2, 3 and 7 for classifying the stillbirth instances into different types of stillbirth.

Clusters are ranked based on their centroid feature scores and the features in the worst clusters are excluded from the feature set for training the classifiers.

### Training the classifiers

Stacked ensemble (SE) classifier as a meta model including the classifiers stacked together in the consecutive layers has been first introduced by Wolpert in 1994 [17]. Its architecture is similar to the neural networks which uses the classifiers in SE instead of neurons in neural networks. SE uses one of normal or re-stacking modes in the stacking operation. Normal stacking mode considers the base classifiers in each layer and feeds the outputs of the classifiers in the current layer as the inputs of the next layer similar to a typical feedforward neural network.

The previous studies have shown that using stacked ensembles could improve the performance of the classification tasks [18, 19]. Therefore, in this study, a new stacked ensemble classifier with normal stacking mode is proposed for stillbirth prediction as shown in Fig. 1 (c).

The proposed SE has two consecutive layers including the same classifiers. The base classifiers in each layer are DT [20], Gradient boosting classifier (GBC) [21], LR, RF [22] and SVM [23] which are trained independently. The main reason of choosing this combination of the base classifiers is their complementary capabilities for classifying dataset with different characteristics and their popularity and promising performance in many previous applications.

The outputs of these base classifiers in the first ensemble layer consist of the first meta feature set which is fed to the second ensemble layer as its input features. The outputs of the base classifiers in the last ensemble layer are concatenated column-wise as the second meta-feature set. The second meta feature set is aggregated by an aggregation rule to classify each input instance. In this study, vote-boosting method is used as the aggregation rule which specifies the instance weights based on the degree of the agreement or disagreement among its assigned labels by the base classifiers in the last ensemble layer. Previous studies have shown the robustness to class-label noise ability for vote-boosting method [24].

After training the classifiers such as DT, GBC, LR, RF and SVM in the first layer, their outputs for each instance are considered as the features F_1i_ (1 ≤ i ≤ 5). F_11_, F_12_, F_13_, F_14_ and F_15_ are the predicted class labels which are outputs of DT, GBC, LR, RF and SVM, respectively. These features are the columns of Meta features1. The Meta features1 are fed into the classifiers of the second layer for training them.

Tuning the hyper-parameters of the classifier is performed with grid-search method. For this purpose, the training dataset is divided into two non-overlapping sets with ratio of 9:1 as training and validation datasets which are used for training the classifiers and assessing the performance of the trained classifiers, respectively.

Our problem is multi class classification which aims to classify data instances into three classes including livebirth, stillbirth before delivery and stillbirth during delivery. Using multi-class classifiers to predict and discriminate three mentioned classes may reduce the accuracy of the model as stated in the previous studies [25]. Therefore, a two-step classifier is proposed and used for classifying livebirth, stillbirth before delivery and stillbirth during delivery as two binary classification tasks. The first step classifies all data records into live birth or stillbirth classes. The data records which are classified into stillbirth class in the first step, are classified into stillbirth before delivery and stillbirth during delivery in the second step. By this way, the multi-class classification task is transformed into two hierarchical binary class classification tasks. Binary classifiers have shown superior performance compared to multi-class classifiers in the previous studies [25].

### Applying the trained classifiers to the test dataset for evaluation and validation

After training the classifiers on the training dataset, they are applied to the test dataset to assess their performance and generalization ability. If the performance of a classifier for predicting the class label of the training dataset is much better than the test dataset, it is overfitted with low generalization ability. To avoid the overfitting of the classifiers and keeping their generalization ability in a high level, the performance of the classifier should be assessed on the test datasets and compared to the classifier performance on the training dataset.

In this study, for the first-step classification task, two classes naming stillbirth as Positive class and live birth as Negative class are considered. The second-step classifiers consider stillbirth during delivery as Positive class and stillbirth before delivery as Negative class. Therefore, two binary-class classification problems should be solved.

For assessing the performance of different classifiers, different performance measures including Accuracy, Precision, Recall, F-Score and Area under Curve (AUC) of the receiver operating characteristics (ROC) should be calculated.

All of the mentioned measures range from 0 (0%) to 1 (100%). The higher values of accuracy, precision, recall, F-Score and AUC are preferred. The average of the measures for the first-step and second-step classifiers is reported.

## Results

The main aims of this study is using machine learning models to predict different types of stillbirth and ranking the features. In this section, the results of feature ranking methods are described at first. Then the performance of the predictive models is reported.

**Fig. 6:**
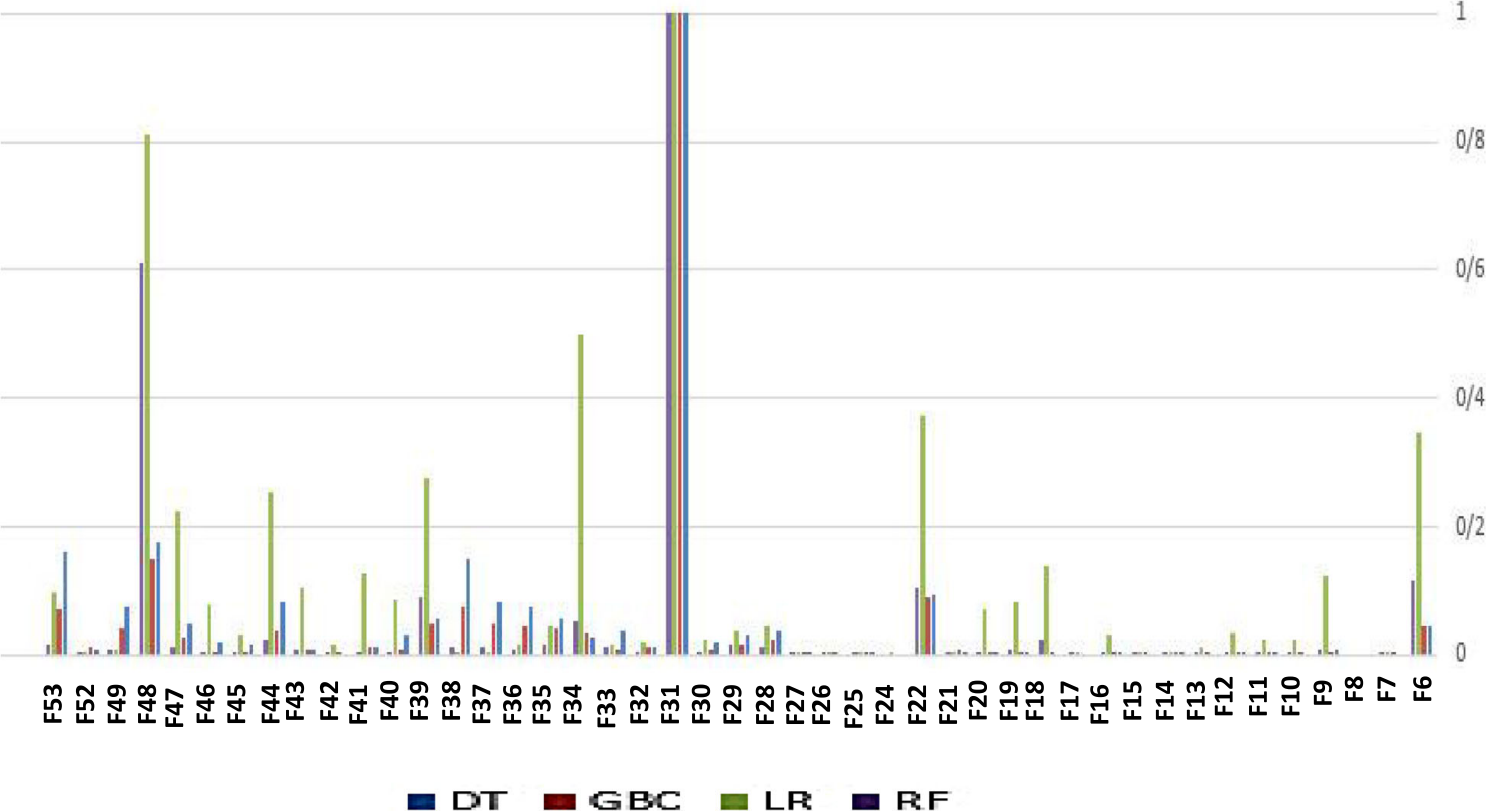
Feature scoring results for discriminating livebirth from stillbirth generated by DT, GBC, LR and RF based on GI, GI, MC and MDA, respectively

For ranking the features, several feature scoring methods are used such as DT, GBC, LR, RF and SVM based on different measures including GI, MDA and MC. Then, the meta feature table (MFT) is clustered to finalized the feature ranking and choose the best clusters of the features for training the models to predict stillbirth cases. Figure 6 shows the feature scores generated by DT and GBC based on GI, LR based on MC and RF based on MDA. For more clarity, other combinations of the feature scoring measures and models are not shown in Fig. 6.

**Fig. 7:**
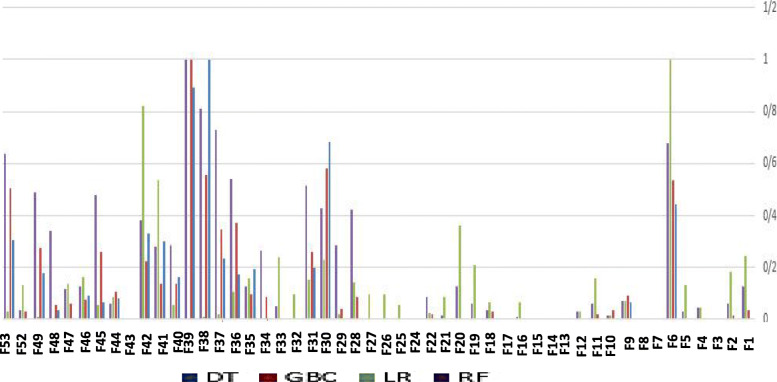
Feature score results for discriminating stillbirth before and during delivery process

For more clarity, all the scores are normalized by min-max normalization method. As shown by Fig. 6, gestational age has obtained the highest score by all the methods DT, GBC, LR and RF. Figure 7 denotes the feature scores for discriminating stillbirth occurring before delivery from stillbirth during delivery.

As shown by Fig. 7, hospital, province, delivery main cause, perinatal abnormality, miscarriage number and maternal age are some important features for classifying stillbirth before and during delivery.

As mentioned in the previous section, the features are clustered based on different feature importance scores and the clusters are ranked based on their corresponding centroid importance scores.

Table 3 lists the results of feature clustering obtained by K-means for different number of the clusters which are the candidates of best clustering results according to the silhouette measure.

**Table 3:** feature clustering results for different number of the clusters

	K	Cluster.Id	Features
Discriminating livebirth from stillbirth	2	1	Gestational age, fetal height
		2	All other features
	4	1	Gestational age
		2	Any pregnancy risk factor, fetal abnormality, delivery risk factors, maternal education, maternal insurance type, hospital type, delivery type
		3	Fetal height
		4	All other features
	6	1	Gestational age
		2	Any pregnancy risk factor, fetal abnormality, delivery risk factors, maternal education, hospital type, delivery type
		3	Gestational diabetes, pre-eclampsia or eclampsia risk factors, placental abruption, Meconium-stained amniotic fluid, labor main cause, fetal gender, maternal insurance type
		4	Fetal height
		5	Province, hospital, region, city, medical science university operator the hospital, cesarean main cause, maternal age
		6	All other features
Discriminating stillbirth before delivery from during it	2	1	Cesarean main cause, gestational age, miscarriage number, fetal abnormality, medical science university operator of the hospital, province, hospital, fetal gender, fetal height, maternal education, delivery main cause, maternal age
		2	All other features
	3	1	Cesarean main cause, gestational age, miscarriage number, fetal abnormality, medical science university operator of the hospital, perinatal gender, perinatal height, maternal education, delivery main cause, maternal age
		2	Province, hospital
		3	All other features
	7	1	Number of the previous deliveries, number of the pregnancies, number of livebirth in the previous pregnancies, hospital type, maternal insurance type
		2	Gestational age, cesarean main cause, medical science university operator of the hospital, fetal height, maternal education, maternal age
		3	Province
		4	Fetal abnormality, fetal gender, delivery main cause
		5	Miscarriage number
		6	Hospital
		7	All other features

Table 3 lists the clusters resulted by K-means for K = 2, 4 and 6 clusters and ranks them based on their centroid importance scores for discriminating livebirth from stillbirth. Moreover, the feature clusters for K = 2, 3 and 7 as the candidates of the best clusters according to silhouette measure are listed in Table 3 for classifying stillbirth before delivery and during it.

Table 4 ranks the feature clusters based on their predictive power.

**Table 4:** ranking the clusters of the features for different number of the clusters

problem	K	Cluster.Id	Rank
Discriminating livebirth from stillbirth	2	CF21	The First rank
		CF22	The second rank
	4	CF41	The first rank
		CF42	The third rank
		CF43	The second rank
		CF44	The fourth rank
	6	CF61	The first rank
		CF62	The third rank
		CF63	The fifth rank
		CF64	The second rank
		CF65	The fourth rank
		CF66	The sixth rank
Discriminating stillbirth before delivery from during it	2	CS21	The first rank
		CS22	The second rank
	3	CS31	The second rank
		CS32	The first rank
		CS33	The third rank
	7	CS71	The sixth rank
		CS72	The fifth rank
		CS73	The second rank
		CS74	The third rank
		CS75	The fourth rank
		CS76	The first rank
		CS77	The seventh rank

Table 5 lists different feature sets fed to the classifiers as their input for stillbirth prediction.

**Table 5:** different feature sets fed to the classifiers as their input variables to predict stillbirth

Feature Set	Included Features
FFS	All features
FSC22	The union of the features belonging to the higher-ranked clusters (CF21 and CS21) while all features are clustered into two clusters
FSC43	The union of the features belonging to the higher-ranked clusters (CF31, CF32, CS41, CS42 and CS43) while all features are clustered into four clusters for the first-step classification and three clusters for the second-step classification
FSC67	The union of the features belonging to the higher-ranked clusters (CF61, CF62, CF63, CF64, CF65, CS71, CS72, CS73, CS74, CS75 and CS76) while all features are clustered into six clusters for the first-step classification and seven clusters for the second-step classification

Table 6 lists the performance measures for different classifiers designed and used in this study for stillbirth prediction.

**Table 6:** comparing the performance of different classifiers for stillbirth prediction in this study

Feature Set	Classifier	Accuracy	Precision	Recall (Sensitivity)	Specificity	F-Score	AUC
FSC22	DT	78.31	66.81	86.79	69.03	75.50	78.31
	LR	76.57	70.38	80.34	70.25	75.03	76.60
	SVM	78.62	65.55	88.76	65.33	75.42	78.69
	GBC	78.54	67.17	86.95	67.18	75.79	78.65
	RF	78.67	67.97	86.49	68.00	76.12	78.67
	SE	79.60	70.85	87.19	71.12	80.03	79.82
FSC43	DT	75.27	71.27	77.50	70.95	74.26	75.24
	LR	80.90	74.75	85.24	74.72	79.66	80.93
	SVM	80.68	71.19	87.88	70.90	78.65	80.59
	GBC	82.02	77.52	85.20	77.48	81.17	82.12
	RF	81.88	75.47	86.59	75.61	80.65	81.91
	SE	89.93	87.10	91.18	87.04	89.90	89.77
FSC67	DT	75.41	76.00	75.13	76.16	75.56	75.32
	LR	73.54	69.76	75.48	69.69	72.51	73.49
	SVM	74.66	83.78	73.96	83.81	78.46	74.35
	GBC	82.37	79.03	84.70	78.84	81.77	82.26
	RF	82.30	75.7	87.24	75.74	81.13	82.17
	**SE**	**90.56**	**88.02**	**91.37**	**88.10**	**90.58**	**90.00**
FFS	DT	73.93	74.90	72.77	74.67	73.82	73.97
	LR	81.74	74.02	86.82	74.11	79.91	81.60
	SVM	68.52	75.10	65.67	74.97	70.07	68.64
	GBC	82.89	76.17	87.34	76.19	81.38	82.77
	RF	80.49	70.02	87.76	70.13	77.89	80.31
	SE	90.55	87.14	92.19	87.06	90.55	89.85

As illustrated by Table 6, the proposed stack ensemble classifier outperforms the compared classifiers for predicting stillbirth cases. The best performance is obtained when the proposed stack ensemble is trained on FSC67 and FFS. It denotes that the features in the lowest ranked clusters of the features resulted by K-means have not significant power to discriminate stillbirth from livebirth cases. Therefore, they can be excluded from the predictor list.

**Fig. 8:**
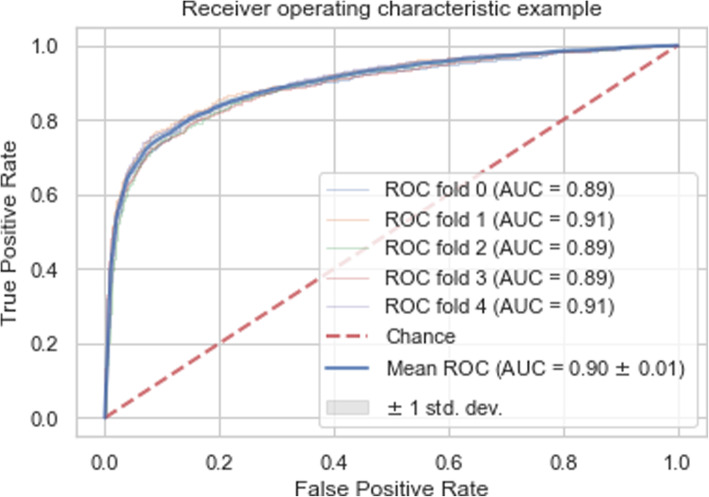
The ROC of the proposed stack ensemble classifier with the best AUC for stillbirth prediction

Figure 8 illustrates the ROC for our proposed stack ensemble which is trained on FSC67 feature set.

As shown by Fig. 8, ROC of the proposed stack ensemble is near to the left and upper axes which demonstrates a very good performance for predicting stillbirth cases.

**Fig. 9:**
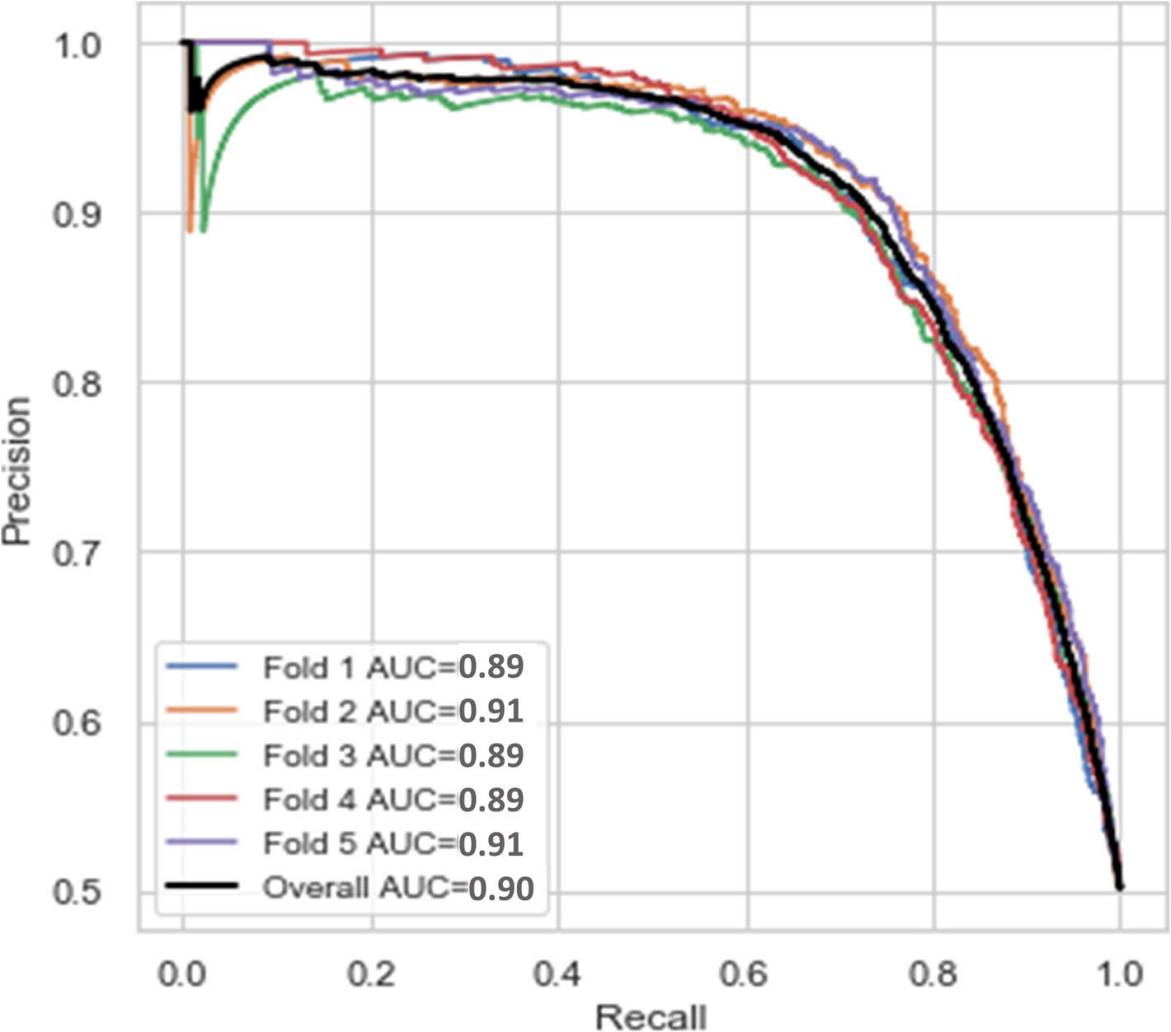
The precision-recall curve for the proposed stack ensemble classifier to predict stillbirth cases

Figure 9 shows the precision-recall curve for the proposed stack ensemble classifier in this study to predict stillbirth cases. The relationship between precision and recall (sensitivity) is shown by precision-recall curve for different cut-off values. Precision-recall curve is less sensitive to the number of data instances belonging to each class in a binary classification. Therefore, it is recommended to show both ROC and Precision-recall curves for assessing the classifier performance.

As shown by Fig. 9, precision-recall curve for each fold of 5-fold C.V. is a zigzag curve. But, all curves are near to the upper and right axis which shows a highly reasonable performance for our proposed stack ensemble classifier in all folds of 5-fold C.V. samples.

The proposed SE is calibrated using isotopic nonparametric calibration method and the calibration score is 0.07. The process is repeated 10,000 times and AUC of SE classifiers using random different training datasets are used as null distribution. Therefore, the significance *p*-value is calculated to assess the specificity of the proposed SE. The obtained *p*-value is 0.0126 which shows the significance of the proposed SE.

## Discussion

The main focus of this study is stillbirth prediction by considering the maternal demographic characteristics, social environmental factors, maternal underlying diseases and risk factors of pregnancy and delivery, maternal related medical history, perinatal descriptors, and information describing the delivery process. The main difference between our study and the previous ones is that their focus has been mainly on early prediction of stillbirth preferably before 24th gestational week. It has been very helpful but could not diagnose the factors which may lead to stillbirth during the delivery process. According to WHO report, about a half of stillbirth cases have occurred during the delivery. In this study, the delivery descriptors are added to the considered feature to predict all stillbirth cases. A two-step classification model is proposed which classifies all birth cases into livebirth and stillbirth in the first step and classifies stillbirth cases into before delivery and during it in the second step.

The proposed SE can be used as a decision support system to predict stillbirth. After training the proposed SE, the input features describing each new case can be fed into the trained proposed SE. Then, the model will assign the new case a class label predicting its outcome as livebirth, stillbirth before delivery and stillbirth during delivery. If the predicted class label for new case is stillbirth before delivery, it is recommended to increase the monitoring and care services during pregnancy for this case. If the new case is classified into stillbirth during delivery, it is suggested that the delivery process is performed carefully using more experiences staff and required instruments, medicine and any other resources.

In this study, two main achievements are obtained:

A new stack ensemble classifier is proposed which outperforms the compared popular classifiers with the average accuracy of 90%. The accuracy of the proposed SE for test dataset is 90% and for training dataset is 90.51% which denotes no overfitting is occurred.

- The features are ranked based on a new proposed method according to their predictive ability for discriminating stillbirth from livebirth and discriminating different types of stillbirth

Although some clinical and anatomical risk factors for early prediction of stillbirth cases are not included in the features considered in this study, the obtained performance for predicting stillbirth by the proposed stack ensemble classifier is surprisingly high.

The most important features in this study for discriminating stillbirth from livebirth are the gestational age and fetal height. The next-rank features are having pregnancy risk factors, perinatal abnormality, delivery risk factors, maternal education, hospital type and delivery type. As mentioned in Table 1, gestational age and fetal height describe perinatal characteristics. Having pregnancy risk factors, delivery risk factors, maternal education and delivery type are related to maternal properties. Hospital type is a health system related factor.

The gestational age and fetal height show the fetal growth status. According to the previous studies, a main factor which may lead to stillbirth is fetal growth restriction [14]. Our main findings confirm this issue, too.

Pregnancy risk factors has been diagnosed as a feature increasing the probability of stillbirth in the previous studies [9] as our findings.

Perinatal abnormality has different categories such as chromosomal abnormalities, pulmonary stenosis, Truncus arteriosus, Coarctation of the aorta and so on. Fetal disorders and malformations has been identified as one of the most important causes of stillbirth in a previous study [26]. Our study results confirm this.

Delivery risk factors and complications has been shown as the predictive features for stillbirth during the delivery process in a previous study [27]. Our experimental results show this, too.

Maternal education is diagnosed in this study as a main factor influencing stillbirth. A previous study has concluded that Stillbirth rates have been increased for mothers with lower levels of education [28].

Hospital type is a feature related to the healthcare system and it is identified as one of the most important features discriminating livebirth from stillbirth in this study. A previous study has confirmed the effect of the healthcare system on increasing or reducing the stillbirth rates [27].

Delivery type (vaginal birth or cesarean) is another important predictor of stillbirth in this study. A previous study has confirmed that the operative delivery, especially Caesarean section, can reduce the rates of stillbirth [29].

The most important predictors for stillbirth cases occurring before and during the delivery process are hospital, province, delivery main cause, perinatal abnormality, miscarriage number and maternal age.

Hospital is a component of a healthcare system and its quality of care can have important effect of controlling stillbirth cases occurring during delivery process. Moreover, different provinces may have different levels of quality of care and it has impact on stillbirth rates.

Delivery main cause is another high-ranked feature diagnosed in this study for differentiating between stillbirth occurring before delivery from stillbirth occurring during it. The previous studies has confirmed that labor complications and placenta problems can lead to stillbirth [29].

Perinatal abnormality is discussed and mentioned above. Therefore, we do not repeat it here.

Miscarriage number is one of the top-features in this study to discriminate stillbirth before from delivery and stillbirth during the labor. The previous studies have found the previous pregnancy loss as an independent factor leading to stillbirth [30].

Maternal advanced age is identified as a high-ranked discriminator for predicting different types of stillbirth cases and it has been recognized as an important predictor leading to stillbirth before delivery in the previous studies, too [31].

The high-ranked predictors of livebirth, stillbirth before delivery and stillbirth during it should be categorized into manageable and unmanageable factors. For manageable factors, the appropriate policies for monitoring and management of them can be adapted to prevent or at least reduce the stillbirth cases.

It is suggested that finding the effectiveness of different policies taken for monitoring and management of high-ranked predictors of stillbirth as a future research direction.

The main limitations of our study are lacking the information of gene expression data and some biomarkers which are important risk factors of occurring stillbirth. Moreover, life style information for pregnant women is not available which may have significant discriminative power between stillbirth and living birth cases.

Therefore, it is proposed to augment IMAN registry dataset with genetic descriptors for maternal and prenatal and life style characteristics for pregnant women to be considered in the future studies for stillbirth prediction.

Moreover, it is suggested to collect data for several years which enables the researchers to find the effect of the environmental factors such as air pollutants, weather condition, epidemic and pandemic situations of infectious diseases, seasonal diseases and other factors on stillbirth and livebirth patterns.

On the other hand, designing and implementing mobile applications to help maternal women for monitoring their health status and preventing from stillbirth due to manageable important factors can be another proposed research opportunity.

## Conclusions

This study proposes machine learning methods for predicting stillbirth as the first task and discriminate stillbirth before delivery from stillbirth occurring during delivery process as the second task. For this purpose, a large national dataset is analyzed including about 1,400,000 records. The proposed stack ensemble classifier for solving this problem outperforms the compared methods with the average accuracy of 90% and AUC of 90%. Moreover, the predictors are ranked based on their predictive power via a new proposed feature scoring method in this study.

In this study, different from the previous studies, a combination of maternal demographic features, clinical history, fetal properties, delivery descriptors, environmental features, healthcare service provider descriptors and socio-demographic features are considered as the input features for stillbirth prediction.

According to the performance of the classifiers, the best feature set to predict stillbirth in our dataset includes gestational age, any pregnancy risk factor, fetal abnormality, delivery risk factors, maternal education, hospital type, delivery type, gestational diabetes, pre-eclampsia or eclampsia risk factors, placental abruption, meconium-stained amniotic fluid, labor main cause, fetal gender, maternal insurance type, fetal height, province, hospital, region, city, medical science university operator the hospital, cesarean main cause, maternal age, number of the previous deliveries, number of the pregnancies, number of livebirth in the previous pregnancies, maternal insurance type, delivery main cause and miscarriage number.

Among them, the top features discriminating livebirth from stillbirth are gestational age and fetal height. Moreover, hospital, province, delivery main cause, perinatal abnormality, miscarriage number and maternal age are identified as the high-ranked features for differentiating between stillbirth before and during delivery.

The average gestational age for stillbirth cases is lower than for livebirth cases. Fetal height as a factor showing fetal growth restrictions is another important feature for discriminating livebirth from stillbirth cases. Perinatal abnormalities and previous miscarriage experience can increase the probability of stillbirth for the current pregnancy. Some provinces can provide limited healthcare services and the rate of stillbirth cases in these provinces are higher than the provinces with higher level of healthcare services. Delivery main cause is an important feature can lead to stillbirth during delivery.

Top features for predicting different types of stillbirth are some maternal and fetal descriptors and healthcare system related factors. They can be divided into manageable and unmanageable groups which adopting the appropriate policies for manageable predictors of stillbirth can reduce the stillbirth rates.

But, our study has main limitation which is the lack of some important risk factors for predicting stillbirth according to the previous studies’ results. It is proposed to augment our dataset with more independent and dependent previously confirmed risk factors of stillbirth and rank all features again in a future study. Moreover, adding life style related risk factors can enable researchers to identify the effect of different life styles on the stillbirth rates.

On the other hand, considering all descriptors of the healthcare systems can be helpful to find the association between the properties of a healthcare system and its related stillbirth rates.

The identified risk factors can be grouped into manageable and unmanageable features. Investigating the effectiveness of different policies for management of the risk factors of stillbirth is a future research opportunity. Finally, implementing mobile applications for monitoring and managing the maternal women who are at higher risk of the future stillbirth is another proposed research direction.

## Supplementary Information

**Additional file 1.** Appendix A: More details about the previous studies related to stillbirth prediction.

**Additional file 2.** Appendix B: The main steps of preprocessing data.

## Data Availability

Our study is a retrospective study of the large population (all stillbirth and living birth cases) registered in Iranian Maternal and neonatal records (IMAN registry) from 2016/04/01 to 2017/01/01. In this study, all births at or beyond 28th gestational week are considered including 1,415,623 live birth and 5,502 stillbirth cases which 4557 and 144 cases are occurred before delivery and during it, respectively. Therefore, administrative permissions are required for access to dataset.

## Abbreviations

AUC: Area Under ROC curve
CFij: The jth cluster resulted by K-means for K = i considering the first-step classification task
CSij: The jth cluster resulted by K-means for K = i considering the second-step classification task
DT: Decision Tree
FFS: Feature set including all features
FSC22: Feature set including the union of the features belonging to the higher-ranked clusters (CF21 and CS21) while all features are clustered into two clusters
FSC43: Feature set including the union of the features belonging to the higher-ranked clusters (CF31, CF32, CS41, CS42 and CS43) while all features are clustered into four clusters for the first-step classification and three clusters for the second-step classification
FSC67: Feature set including the union of the features belonging to the higher-ranked clusters (CF61, CF62, CF63, CF64, CF65, CS71, CS72, CS73, CS74, CS75 and CS76) while all features are clustered into six clusters for the first-step classification and seven clusters for the second-step classification
GBC: Gradient Boosting Classifier
HIV: Human immunodeficiency viruses
IUGR: Intrauterine growth restriction
LR: Logistic Regression
RF: Random Forest
ROC: Receiver Operating Characteristics Curve
SVM: Support Vector Machines
VDRL: Venereal Disease Research Laboratory test
SE: Stack Ensemble
