# Supplementary material for: Proposing a machine-learning based method to predict stillbirth before and during delivery and ranking the features: nationwide retrospective cross-sectional study
Source: BMC Pregnancy Childbirth. 2021 Mar 12;21:202. doi: 10.1186/s12884-021-03658-z (PMC7953639; doi:10.1186/s12884-021-03658-z)
Supplement: Supplementary file 1 — Additional file 1. Appendix A: More details about the previous studies related to stillbirth prediction. [file 12884_2021_3658_MOESM1_ESM.docx]

Appendix A: More details about the previous studies related to stillbirth prediction

**The features considered in the previous studies for stillbirth prediction**

Nulliparity, advanced age and obesity have been diagnosed as some independent risk factors of stillbirth [1]. Birth weight has been diagnosed as a risk factor of stillbirth, too [2].

According to a previous study, some important risk factors leading to stillbirth have been prior stillbirth, the etiology of the prior stillbirth, fetal growth restriction, gestational age of the prior stillbirth, and race [3].

Fetal growth restriction has been diagnosed as a main risk factor of stillbirth occurrence in the previous studies [4]. Moreover, small-for-gestational age (SGA) feature has been determined as a good predictor for high risk of stillbirth [5].

Maternal comorbidity, place of residence, maternal occupation, parity, bleeding in pregnancy, fetal presentation and fetal growth rate have been analyzed for early prediction of stillbirth [6].

A previous study has considered maternal serum placental growth factor (PlGF), maternal factors, fetal biometry and uterine artery pulsatility index (UtA-PI) to predict stillbirth [7].

Maternal weight, race, smoking, chronic hypertension, systemic lupus erythematosus/ antiphospholipid syndrome, diabetes, and previous stillbirth have been recognized as the main risk factors for occurring stillbirth in the current pregnancy [8].

A previous cohort study has concluded that maternal age, black race, nulliparity, body mass Index (BMI), smoking, chronic hypertension and pre-gestational diabetes are the main risk factors of stillbirth [9].

Amar et al. have considered different features for stillbirth prediction including BMI, Papp-a, age, smoking status, living country, annual income, educational level, mothers living alone, drug abuse, nulliparous, previous SGA, previous stillbirth, previous pre-eclampsia, gestational age, antiphosholipid syndrome, SLE, pregnancy hypertension, essential hypertension, GDM, early fetal growth and pre-eclampsia [10].

Some researchers have analyzed the metabolomics features extracted from the first-trimester maternal serum on 180 cases to predict stillbirth before 32 weeks of maternity. They have concluded that considering metabolomics and clinical features together can increase the prediction AUC [11].

Another study has analyzed the features including the maternal socio-demographic features, chronic medical conditions, obstetric complications and family history in both the current and previous pregnancy for stillbirth prediction in the current pregnancy [12].

**The analytical methods in the previous studies for predicting stillbirth**

The previous studies have used univariate [13] and/or multivariate statistical analysis [8, 10] and machine learning classifiers [12] for stillbirth prediction.

Some previous studies have used multi-variate logistic regression for stillbirth prediction [5-8].

A previous study has used and trained some machine learning models for stillbirth prediction including regularized logistic regression (RLR), decision trees (DT), random forest, extreme gradient boosting (XGBoost), and a multilayer perceptron neural network (MLP) with XGBoost has outperformed the compared models [12]. Backward stepwise logistic regression has been used for stillbirth prediction and risk scoring [9]. Another study has used logistic regression for stillbirth prediction among pregnant women with obesity [10].

**The analyzed dataset characteristics in the previous studies for stillbirth prediction**

A previous study has conducted a prospective screening study of 70,003 singleton pregnancies leading to 268 stillbirth [7]. For this purpose, the features describing placental growth have been extracted between 19^th^ and 24^th^ gestational week for stillbirth prediction [7].

A retrospective cohort study has considered 6,573 pregnancies in a tertiary level of healthcare in Nigeria from January 2010 to December 2013 for early diagnosis of high risk stillbirth cases [6]. Another study has assessed 57,170 pregnancies in which 319 stillbirth cases have been occurred [5].

A prospective screening study have focused on 113,415 singleton pregnancies between 11^th^ to 14^th^ and 19^th^ to 25^th^ gestational week for discriminating between stillbirth and live birth cases [8].

A cohort study has considered dataset of 64,173 singleton pregnancies undergoing second trimester anatomic survey from 1999-2009 [9].

A Cohort study of 979,912 singleton pregnancies including 1672 stillbirth has been collected and analyzed in a previous study [2].

Amark et al. have considered stillbirth after 28th gestational week in singleton pregnancies for women with obesity [10]. They have considered cohort data which has been registered from 2006 until 2015 with the Swedish Medical Birth Register and the Swedish Register of Total Population. 282 stillbirths among 45859 pregnancies with obesity have been occurred [10].

Neogi et al. have analyzed the effect of the healthcare system features to stillbirth during the delivery. They have concluded that a good health system can prevent many of stillbirth cases occurring during the labor process. Moreover, they have shown that increased maternal age, previously preterm births, smoking, and history of injury or physical violence during pregnancy can lead to stillbirth during delivery process [14].

A previous cohort study has collected data for 947,025 livebirths and 5,788 stillbirths in Western Australia from 1980 to 2015 [12].

Appendix References:

1. Smith, G.C., *Predicting antepartum stillbirth.* Curr Opin Obstet Gynecol, 2006. **18**(6): p. 625-30.

2. Iliodromiti, S., et al., *Customised and Noncustomised Birth Weight Centiles and Prediction of Stillbirth and Infant Mortality and Morbidity: A Cohort Study of 979,912 Term Singleton Pregnancies in Scotland.* PLOS Med, 2017. **14**(1): p. e1002228.

3. reddy, U.M., *Prediction and Prevention of Recurrent Stillbirth.* Obstetrics and Gynecology, 2007. **110**(5): p. 1151-1164.

4. Bukowski, R., *Stillbirth and fetal growth restriction.* Clinical Obstetrics and Gynecology, 2010. **53**(3): p. 673-680.

5. Trudell, A.S., et al., *Stillbirth and the small fetus: use of a sex-specific versus a non-sex-specific growth standard.* Journal of Perinatology, 2015. **35**(8): p. 566-9.

6. Kayode, G.A., et al., *Predicting stillbirth in a low resource setting.* BMC Pregnancy Childbirth, 2016. **16**.

7. Aupont, J.E., et al., *Prediction of stillbirth from placental growth factor at 19-24 weeks.* Ultrasound Obstetrics and Gynecology, 2016. **48**(5): p. 631-635.

8. Yerlikaya, G., et al., *Prediction of stillbirth from maternal demographic and pregnancy characteristics.* Ultrasound Obstetrics and Gynecology, 2016. **48**(5): p. 607-612.

9. Trudell, A.S., et al., *A stillbirth calculator: Development and internal validation of a clinical prediction model to quantify stillbirth risk.* PLOS One, 2017. **12**(3): p. e0173461.

10. Amark, H., M. Westgren, and M. Persson, *Prediction of stillbirth in women with overweight or obesity-A register-based cohort study.* PLOS One, 2018. **13**(11): p. e0206940.

11. Bahado-Singh, R.O., et al., *First-trimester metabolomic prediction of stillbirth.* The Journal of Maternal-Fetal & Neonatal Medicine, 2019. **32**(20): p. 3435-3441.

12. Malacova, E., et al., *Stillbirth risk prediction using machine learning for a large cohort of births from Western Australia, 1980–2015.* Scientific Reports, 2020. **10**: p. 5354.

13. Flenady, V., et al., *Major risk factors for stillbirth in high-income countries: a systematic review and meta-analysis.* Lancet, 2011. **377**(9774): p. 1331–1340.

14. Neogi, S.B., et al., *Risk factors for stillbirths: how much can a responsive health system prevent?* BMC Pregnancy and Childbirth, 2018. **18**.

15. Han, J., M. Kamber, and J. Pei, *Data mining: Concepts and Techniques*. 2012: Morgan Kauffmann.

16. Rousseeuw, P.J., *Silhouettes: a graphical aid to the interpretation and validation of cluster analysis* Journal of Computational and Applied Mathematics, 1987. **20**: p. 53-65.
