# Supplementary material for: Proposing a machine-learning based method to predict stillbirth before and during delivery and ranking the features: nationwide retrospective cross-sectional study
Source: BMC Pregnancy Childbirth. 2021 Mar 12;21:202. doi: 10.1186/s12884-021-03658-z (PMC7953639; doi:10.1186/s12884-021-03658-z)
Supplement: Supplementary file 2 — Additional file 2. Appendix B: The main steps of preprocessing data. [file 12884_2021_3658_MOESM2_ESM.docx]

Appendix B: The main steps of preprocessing data

- **Data Sampling**

To avoid over-fitting the models and measuring the generalization ability, dataset should be partitioned into two non-overlapping sets naming training and test datasets. Training dataset is used to train the classifiers and test dataset is fed to the trained models to evaluate their performance. Several different sampling methods have been proposed and used for data sampling in the previous studies such as sampling with or without replacement, oversampling, under-sampling and so on. A commonly used and popular method for sampling from datasets is K-fold Cross Validation (C.V.) which partitions dataset into K equal-sized non-overlapping partitions and trains the classifier K times [15]. Each time, the classifier is trained on the instances belonging to (K-1) partitions and assessed on the instances of the remaining partition.

Dataset is extremely imbalanced for stillbirth and livebirth classes as the minority and majority classes, respectively. Therefore, stratified 5-fold C.V. as the sampling method is used in this study.

- **Missing value imputation**

Since our dataset has a large number of instances, simple and fast missing value imputation methods are used in this study.

There are four different feature types in this study including binary, ordinal, numeric and nominal features. For ordinal and numeric features, the missing values are replaced with the median of the feature because of robustness of median against noise and outliers. Binary and nominal features use th e mode (maximum frequency) of the feature for missing value imputation.

The features having more than 5% missing values, are excluded from the study and the missing values for the remaining features are imputed using the mentioned methods. The data instances having more than 10% missing values are excluded from the study.

- **Nominal variable conversion**

Nominal and categorical variables are converted into dummy binary variables in this study according to the previously proposed methods for converting nominal variables [15].

- **Normalization**

The ordinal and numerical features are normalized with min-max normalization method as proposed in the previous studies [15] to avoid dominating the feature with smaller range of variation with the features having larger range of variation. Min-max normalization of the features is calculated as Eq. (1):

$$F_{i}=\frac{F_{i}-min(F_{i})}{({max(F}_{i})-{min(F}_{i}))} (1)$$

Where F_i_ is the i^th^ feature and Min(F_i_) and Max(F_i_) are the minimum and maximum values of F_i_ column, respectively.

Appendix B: The equations of MDA and GI

MDA is calculated as Eq. (2):

$MDA\left( F_{p}.{Classifier}_{s} \right)=\frac{\sum_{c=1}^{n} (Accuracy({Classifier}_{s})-Accuracy({Classifier}_{s-F_{p}})}{n}$ (2)

Where F_p_ indicates the p^th^ input feature of our dataset, Classifier_s_ is the s^th^ classifier used in this study trained on all input features, Classifier_s-Fp_ is the s^th^ classifier trained on all input features excluding the p^th^ feature. n is the number of iterations which should be at least 30 iterations.

GI is calculated as Eq. (3):

$$Gini Index(F_{p})=1-\sum_{j=1}^{2} p_{j|F_{p}}^{2} (3)$$

Where pj is the probability that an arbitrary data instance of training dataset belongs to class *C_j_*.

Appendix C: Other equations used in this study

Distance function used in this study is the Euclidean distance which can be calculated as Eq.(4):

$$Distance\left( R_{i}R_{j} \right)=\sqrt{\sum_{p=1}^{m} {(C_{ip}-C_{jp})}^{2}} (4)$$

Where m is the number of MFT columns, C_i,p_ and C_j,p_ denote the p^th^ MFT column corresponding to p^th^ feature score for MFT rows corresponding to R_i_ and R_j_.

Silhouette is calculated based on intra cluster tightness and inter cluster separation as Eq.(5)-(8) [16]:

$silhouette=\frac{\sum_{j=1}^{n} s(j)}{n}$ (5)

$$s\left( j \right)=\frac{b\left( j \right)-a\left( j \right)}{\max\left( a\left( j \right).b\left( j \right) \right)} (6)$$

$$a\left( j \right)=the average dissimilarity of jth instance to all other objects in the same cluster (7)$$

$b\left( j \right)=the average dissimilarity of jth instance to all other objects in another cluster (8)$

The formula of normal stacking mode is shown as Eq. (9):

$$f_{n}\left( xV \right)= V_{nk}\left( f_{n-1}\left( xV_{n-11} \right) f_{n-1}\left( xV_{n-12} \right) f_{n-1}\left( xV_{n-1D_{n-1}} \right) \right) (9)$$

Where N is the size of hidden layers, n indicates the n^th^ layer of the N, x denotes a sample of a dataset, V presents a vector holding the neurons (the base classifiers), D is the number of hidden neurons through the n^th^ hidden layer and finally, k is the k^th^ neuron in the n^th^ layer.

Accuracy calculates how many data records are classified correctly as Eq. (10):

|  | $Accuracy=\frac{TP+TN}{N}$ | (10) |
| --- | --- | --- |

where TP and TN are true positive and true negative data records and N is the number of all data records. TP and TN denote the number of stillbirth and live birth cases which are classified correctly with the classifier, respectively. Precision or positive predictive value denotes as Eq. (11) measuring for how many instances assigned positive label by the classifier, the real class label is positive, too. Recall or sensitivity measures how many data records belonging to positive class are classified correctly as Eq. (12):

|  | $Precision=\frac{\mathrm{TP}}{TP+FP}$ | (11) |
| --- | --- | --- |

|  | $Recall=\frac{\mathrm{TP}}{TP+FN}$ | (12) |
| --- | --- | --- |

where FP and FN are false positive and false negative data records.

F_Score is the harmonic average of Precision and Recall and is calculated as Eq. (13):

$$F-Score=2*\frac{Precision*Recall}{Precision+Recall} (13)$$

Appendix References:

1. Smith, G.C., *Predicting antepartum stillbirth.* Curr Opin Obstet Gynecol, 2006. **18**(6): p. 625-30.

2. Iliodromiti, S., et al., *Customised and Noncustomised Birth Weight Centiles and Prediction of Stillbirth and Infant Mortality and Morbidity: A Cohort Study of 979,912 Term Singleton Pregnancies in Scotland.* PLOS Med, 2017. **14**(1): p. e1002228.

3. reddy, U.M., *Prediction and Prevention of Recurrent Stillbirth.* Obstetrics and Gynecology, 2007. **110**(5): p. 1151-1164.

4. Bukowski, R., *Stillbirth and fetal growth restriction.* Clinical Obstetrics and Gynecology, 2010. **53**(3): p. 673-680.

5. Trudell, A.S., et al., *Stillbirth and the small fetus: use of a sex-specific versus a non-sex-specific growth standard.* Journal of Perinatology, 2015. **35**(8): p. 566-9.

6. Kayode, G.A., et al., *Predicting stillbirth in a low resource setting.* BMC Pregnancy Childbirth, 2016. **16**.

7. Aupont, J.E., et al., *Prediction of stillbirth from placental growth factor at 19-24 weeks.* Ultrasound Obstetrics and Gynecology, 2016. **48**(5): p. 631-635.

8. Yerlikaya, G., et al., *Prediction of stillbirth from maternal demographic and pregnancy characteristics.* Ultrasound Obstetrics and Gynecology, 2016. **48**(5): p. 607-612.

9. Trudell, A.S., et al., *A stillbirth calculator: Development and internal validation of a clinical prediction model to quantify stillbirth risk.* PLOS One, 2017. **12**(3): p. e0173461.

10. Amark, H., M. Westgren, and M. Persson, *Prediction of stillbirth in women with overweight or obesity-A register-based cohort study.* PLOS One, 2018. **13**(11): p. e0206940.

11. Bahado-Singh, R.O., et al., *First-trimester metabolomic prediction of stillbirth.* The Journal of Maternal-Fetal & Neonatal Medicine, 2019. **32**(20): p. 3435-3441.

12. Malacova, E., et al., *Stillbirth risk prediction using machine learning for a large cohort of births from Western Australia, 1980–2015.* Scientific Reports, 2020. **10**: p. 5354.

13. Flenady, V., et al., *Major risk factors for stillbirth in high-income countries: a systematic review and meta-analysis.* Lancet, 2011. **377**(9774): p. 1331–1340.

14. Neogi, S.B., et al., *Risk factors for stillbirths: how much can a responsive health system prevent?* BMC Pregnancy and Childbirth, 2018. **18**.

15. Han, J., M. Kamber, and J. Pei, *Data mining: Concepts and Techniques*. 2012: Morgan Kauffmann.

16. Rousseeuw, P.J., *Silhouettes: a graphical aid to the interpretation and validation of cluster analysis* Journal of Computational and Applied Mathematics, 1987. **20**: p. 53-65.
